# Retinal Changes in Chronic Kidney Disease: A Spectral-Domain Optical Coherence Tomography Study Correlated with Biochemical Parameters

**DOI:** 10.22336/rjo.2026.38

**Published:** 2026

**Authors:** Sanagama Bhargav, Priyanka Pantola, Avadhesh Oli

**Affiliations:** 1 Post Graduate Department of Ophthalmology, Command Hospital Air Force, Bangalore, India

**Keywords:** chronic kidney disease, optical coherence tomography, retinal nerve fibre layer, ganglion cell complex, choroidal thickness, CKD = Chronic Kidney Disease, OCT = Optical coherence tomography, SD-OCT = spectral-domain optical coherence tomography, BUN = blood urea nitrogen, CRT = Central retinal thickness, CCT = Central choroidal thickness, RNFL = retinal nerve fibre layer, GCC = ganglion cell complex, GFR = glomerular filtration rate, Hb = hemoglobin, FBS = fasting blood sugar, PPBS = post-prandial blood sugar, FFA = fundus fluorescein angiography, ILM = internal limiting membrane, EDI = Enhanced depth imaging, GCA = Ganglion cell analysis, RAS = renin-angiotensin system

## Abstract

**Background:**

Chronic Kidney Disease (CKD) is a significant public health problem associated with considerable morbidity and mortality. It is known to cause retinal vascular changes and retinopathy that reflects changes occurring in the kidneys. Optical coherence tomography (OCT) is a promising biomarker for assessing retinal changes associated with CKD. This study was conducted to evaluate retinal alterations in patients with CKD using spectral-domain optical coherence tomography (SD-OCT), correlate these findings with biochemical parameters to facilitate early screening and timely detection, and assess disease progression.

**Materials and methods:**

This single-centre, cross-sectional study included 60 eyes of 60 patients with stage 3, 4, or 5 CKD. Data collected included age, sex, glycaemic status, serum electrolytes, blood urea nitrogen (BUN), and serum creatinine. Fundus examination and various OCT parameters were measured. Central retinal thickness (CRT) and central choroidal thickness (CCT) were measured manually at the subfoveal region. Automated segmentation was used to measure retinal nerve fibre layer (RNFL) and ganglion cell complex (GCC) thicknesses across all quadrants and sectors using SD-OCT. Descriptive and inferential statistical analyses were applied to assess significance.

**Results:**

Most patients were in Stage 3 CKD, with the majority having a disease duration of 1-3 years. Mean retinal thickness reduced from stage 3 to 5 with no statistical significance, while choroidal thickness remained preserved. Inferior RNFL thickness declined significantly with worsening CKD, whereas temporal RNFL and GCC showed borderline significance. Retinal parameters did not differ by CKD duration. Elevated HbA1c was associated with increased retinal thickness and thicker inferior RNFL. Both hypernatremia and hyperkalemia were associated with reduced retinal thickness. A higher glomerular filtration rate (GFR) was positively correlated with inferior RNFL thickness.

**Discussion:**

The findings of this study suggest that chronic kidney disease is associated with progressive neuroretinal changes, particularly thinning of the inferior retinal nerve fibre layer, which may reflect underlying microvascular and neurodegenerative processes occurring in CKD. The preservation of choroidal thickness despite declining renal function indicates that neuroretinal alterations may precede detectable choroidal changes. The observed associations between OCT parameters and biochemical markers, including HbA1c, serum sodium, potassium, and GFR, further support the influence of systemic metabolic and renal status on retinal structure.

**Conclusion:**

The study highlights that chronic kidney disease is associated with neuroretinal alterations that become more pronounced as disease severity increases. Optical Coherence Tomography has emerged as an important biomarker for evaluating these retinal changes. Further, by correlating OCT findings with biochemical parameters, we can evaluate the impact of systemic parameters in CKD on the retina. Therefore, OCT is an important tool in CKD that helps with screening, monitoring, and assessing disease progression.

## Introduction

Chronic kidney disease (CKD) is defined as an abnormality of renal structure and function of more than 3 months' duration, with a reduced glomerular filtration rate (GFR) below 60 mL/min/1.73 m2 and associated with other markers of kidney damage, such as albuminuria and urinary sediment abnormalities. It is classified according to CGA staging (Cause, GFR category, and Albuminuria category) [1]. There are 5 GFR categories (G1, G2, G3a, G3b, G4, G5) and 3 albuminuria categories (A1, A2, A3) [2].

With approximately 700 million people affected worldwide, CKD is a significant public health issue, particularly in developing countries. In India, its prevalence is about 13.24% [3,4]. CKD is associated with significant morbidity and mortality due to reduced GFR contributing to systemic complications like anaemia, malnutrition, bone mineral abnormalities, cardiovascular diseases and electrolyte disturbances. Ocular conditions associated with CKD include cataracts, subconjunctival concretions, and retinal and choroidal changes, such as microvascular and diabetic retinopathy, retinal hemorrhage, and macular degeneration [5]. Retinopathy is also more severe in patients with lower GFR and higher CKD stage (Stages 3-5) [5].

The retina is unique in that it enables direct, non-invasive monitoring of the central nervous system and microvascular perfusion. Its neural connection is established by the fact that the optic vesicle and diencephalon outgrowth give rise to the retina. Furthermore, several studies have demonstrated subtle neurosensory retinal changes in neurodegenerative disorders, particularly Alzheimer’s disease [6-9]. Anatomical and physiological features of the brain, such as high perfusion volume due to low vascular resistance, make it similar to the kidney [6]. Such brain-kidney similarities have been elucidated by various studies that correlate cognitive disorders with chronic kidney disease [7-9]. Considering the above correlations, this study was undertaken to explore the impact of renal pathology, such as chronic kidney disease, on retinal neural microstructure.

Retinal vascular changes mirror those in other organs, such as the heart and kidneys. The renal podocyte is analogous to the vascular pericyte, both of which possess a high surface area with interdigitating foot processes. Various studies have linked retinopathy and nephropathy [10-14]. Earlier, fundus photography substantiated this, but optical coherence tomography (OCT) is now a promising biomarker for studying these retinal changes. OCT uses low-coherence interferometry to image the retina at 10- micron depth resolution. Enhanced depth imaging improves choroidal visualization and thickness measurement.

There is limited research on the correlation between CKD and retinochoroidal alterations. This study, by incorporating both retinal and biochemical parameters, provides a comprehensive assessment of the impact of this systemic condition on ocular well-being in patients with CKD. This cross-sectional study examined retinal alterations in various stages of CKD to facilitate early ocular screening in CKD management. This preventive measure is essential considering the long-term cost and the challenges associated with dialysis and renal transplantation.

## Materials and methods

This was a single-centre, cross-sectional study. The Institutional Ethics Committee approved the study protocol, and the study adhered to the tenets of the Declaration of Helsinki. Informed consent was obtained from all patients. A total of 60 subjects diagnosed as stage 3, 4, or 5 CKD were recruited.

The inclusion criteria were volunteers aged ≥ 18 years with CKD stages 3, 4, or 5. The exclusion criteria were: (1) other choroidal and vitreoretinal diseases; (2) medications known to affect choroidal thickness; (3) active intraocular inflammation; (4) vitreoretinal or cataract surgery within the past 6 months; 5) spherical equivalent refractive error ≥ -6 DS in myopes and ≥ +3 DS in hypermetropes; 6) poor-quality OCT images due to significant media opacity.

The demographic details of the patients were noted. Laboratory parameters that were recorded include hemoglobin (Hb), fasting blood sugar (FBS), post-prandial blood sugar (PPBS), HbA1c, serum sodium, potassium, albumin, and GFR. All subjects underwent a comprehensive ophthalmic examination, including visual acuity testing, intraocular pressure measurement using a non-contact tonometer, slit-lamp examination to assess lens status, and indirect ophthalmoscopy. Digital fundus fluorescein angiography (FFA) was performed as needed.

All SD-OCT measurements were obtained using the same device, the Zeiss Cirrus HD-OCT 6000 (Carl Zeiss Meditec, Inc, 5300 Central Parkway, Dublin, CA 94568 USA), to capture high-definition retinal and choroidal images. Central retinal thickness (CRT) was calculated using automated software from the internal limiting membrane (ILM) to the Bruch’s membrane edge. The choroid was imaged in Enhanced depth imaging (EDI) mode at the foveal centre. EDI automatically adjusted for the position of the choroid closer to the zero-delay line to enhance visualization of the choroidal-scleral interface. Choroidal thickness was measured manually using high-precision software calipers at right angles from the outer rim of the retinal pigment epithelium to the choroidoscleral margin.

**Fig. 1:**
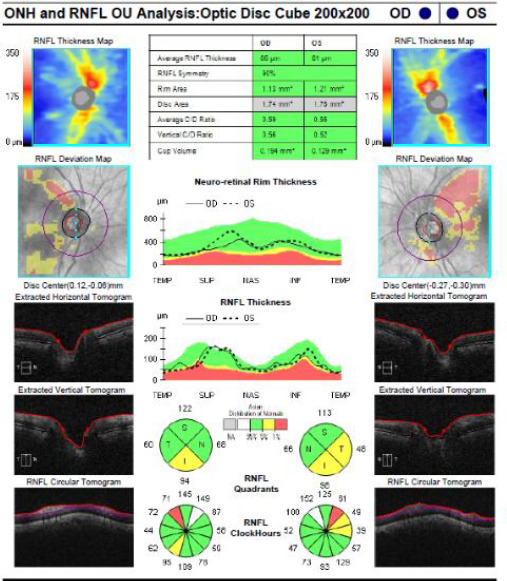
Automated software quantification of the retinal nerve fibre layer (RNFL) thickness in various quadrants and clock hours using spectral domain OCT (SD-OCT)

**Fig. 2:**
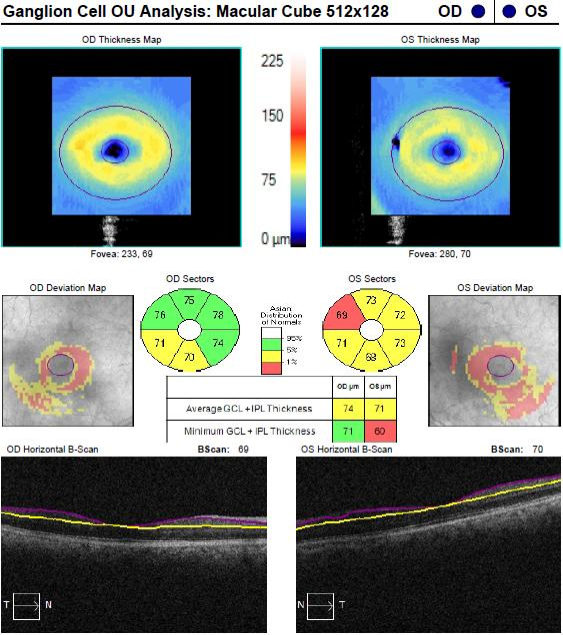
Ganglion cell analysis (GCA) showing automated software quantification of ganglion cell layer (GCL) and inner plexiform layer (IPL) in various sectors around the fovea

Retinal nerve fibre layer (RNFL) thickness was calculated using automated software in four quadrants centered over the optic nerve. Similarly, Ganglion cell analysis (GCA) was done using automated software quantification in various sectors around the fovea (Fig. 1, 2). Only data from one eye per patient, randomly selected, were included in the statistical analysis. Statistical analysis was performed using SPSS (Statistical Package for Social Sciences) version 21 (IBM Corporation, NY, USA).

## Results

**Fig. 3:**
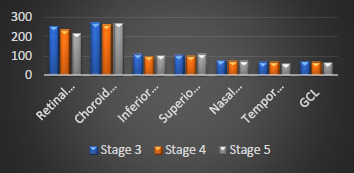
Association between various retinal morphological parameters and stages of CKD

Table 1 shows the demographic and clinical profile of the study participants. Most patients were in Stage 3 CKD, with the majority having a disease duration of 1-3 years. Table 2 summarizes various retinal parameters across CKD stages 3, 4, and 5 using a one-way ANOVA test. It shows selective, non-uniform retinal involvement that advances with renal disease (Fig. 3).

**Table 1:** Demographic and Clinical Profile of Study Participants with Chronic Kidney Disease (CKD)

Parameter	Category	Frequency (n) (Total 60)	Percentage (%)
Age (years)	40-50 years	6	10.00%
	51-60 years	15	25.00%
	61-70 years	23	38.33%
	71-80 years	13	21.67%
	>80 years	3	5.00%
Gender	Male	43	71.67%
	Female	17	28.33%
Duration of CKD	<1 year	21	35.00%
	1-3 years	24	40.00%
	>3 years	15	25.00%
Stage of CKD	Stage 3	42	70.00%
	Stage 4	10	16.67%
	Stage 5	8	13.33%
Phakic Status	Phakic	43	71.67%
	Pseudophakic	17	28.33%

**Table 2:** Association between various retinal morphological parameters and stages of CKD

Retinal Morphological Parameters (Mean values)	CKD			ANOVA Test value	P- value
	Stage 3	Stage 4	Stage 5		
Retinal thickness (CRT) (µm)	250.26 ± 43.98	233.2 ± 26.24	216.375 ± 27.97	2.77	0.07
Choroidal thickness (µm)	269.47 ± 50.93	259.7 ± 51.04	265.87 ± 58.6	0.146	0.86
Inferior RNFL (µm)	110.24 ± 11.97	98.8 ± 10.12	103.375 ± 9.4	4.64	0.01
Superior RNFL (µm)	104.8 ± 12.63	99.5 ± 11.4	110.25 ± 12.25	1.68	0.19
Nasal RNFL (µm)	75.88 ± 7.8	75 ± 4.2	74.5 ± 4.75	0.166	0.84
Temporal RNFL (µm)	68.38 ± 9.23	69.6 ± 5.08	60.8 ± 6.625	3.0775	0.05
Ganglion cell analysis (GCA) (µm)	70.93 ± 4.65	68.4 ± 3.72	67.125 ± 5.625	2.96	0.05

Mean retinal thickness decreased progressively from CKD Stage 3 to Stage 5. However, this did not reach statistical significance (p = 0.07). Choroidal thickness remained comparable across all CKD stages, with no significant difference (p = 0.86), indicating relative structural preservation.

A significant reduction in inferior retinal nerve fibre layer (RNFL) thickness was observed (p = 0.01), indicating progressive RNFL thinning with advancing CKD. However, the lowest values were observed in stage 4, with a partial increase in stage 5, suggesting a non-linear pattern. Superior and nasal RNFL thickness did not differ significantly between stages (p = 0.19 and 0.84, respectively). Temporal RNFL thickness showed a borderline significant difference (p = 0.05), reflecting neuroretinal involvement with CKD severity.

Ganglion cell complex (GCC) thickness decreased progressively with increasing CKD severity, though the difference was borderline significant (p = 0.05). These findings suggested that as CKD progresses, certain retinal layers might be more susceptible than others.

We further examined the relationship between retinal morphological parameters and CKD duration (Table 3). No significant differences were observed in mean retinal thickness, choroidal thickness, RNFL, or GCL thickness across CKD duration groups (< 1 year, 1-3 years, and >3 years). Variations in mean values were not statistically significant, indicating other factors might contribute to retinal alterations in CKD.

**Table 3:** Association between retinal morphological parameters and duration of CKD

Retinal Morphological Parameters (Mean values)	< 1 year (n=21)	1-3 years (n=24)	> 3 years (n=15)	ANOVA Test value	P- value
Retinal thickness (CRT) (µm)	245.96 ± 39.52	243.3 ± 39.62	242.97 ± 39.77	0.03	0.966
Choroidal thickness (µm)	263.33 ± 50.89	267.60 ± 51.94	269.15 ± 52.45	0.06	0.937
Inferior RNFL (µm)	107.8 ± 12.24	107.86 ± 12.00	107.14 ± 11.67	0.01	0.98
Superior RNFL (µm)	103.8 ± 12.48	104.40 ± 12.90	104.50 ± 13.00	0.02	0.98
Nasal RNFL (µm)	75.54 ± 6.76	76.01 ± 6.57	75.76 ± 6.59	0.03	0.97
Temporal RNFL (µm)	68.37 ± 8.11	67.83 ± 8.54	67.38 ± 8.41	0.06	0.94
Ganglion cell analysis (GCA) (µm)	70.14 ± 4.63	69.86 ± 4.79	69.87 ± 4.81	1.72	0.18

Table 4 highlights key associations between biochemical markers and OCT parameters. Higher HbA1c levels correlated with increased retinal thickness and thicker inferior RNFL. Electrolyte imbalances also affected retinal morphology; whereas hypernatremia was associated with reduced retinal thickness and significantly thinner inferior and nasal RNFL, hyperkalemia was associated with reduced retinal thickness. Additionally, higher GFR was positively correlated with inferior RNFL thickness.

**Table 4:** Correlation of OCT Findings with Biochemical Parameters

Biochemical Parameter	Retinal Thickness (CRT)	Choroidal Thickness	RNFL Inferior	RNFL Superior	RNFL Nasal	RNFL Temporal	GCC (Ganglion Cell Complex)
HbA1c	0.26 (P = 0.045, significant)	-0.05 (P > 0.6)	0.28 (P = 0.03, significant)	-0.03 (P > 0.7)	0.19 (P = 0.14)	0.16 (P = 0.22)	-0.11 (P = 0.4)
Hb	-0.03 (P > 0.7)	-0.19 (P = 0.14)	0.17 (P = 0.19)	0.04 (P > 0.7)	-0.02 (P > 0.8)	-0.11 (P = 0.4)	-0.08 (P = 0.5)
BUN	-0.08 (P = 0.5)	-0.03 (P > 0.7)	-0.21 (P = 0.09, significant)	-0.01 (P > 0.9)	0.07 (P = 0.6)	-0.07 (P = 0.6)	0.05 (P = 0.7)
Serum Creatinine	-0.13 (P = 0.32)	0.06 (P = 0.6)	-0.25 (P = 0.05, significant)	0.03 (P > 0.7)	0.003 (P > 0.9)	-0.18 (P = 0.16)	-0.07 (P = 0.6)
Serum Sodium	-0.25 (P = 0.05, significant)	-0.15 (P = 0.24)	-0.29 (P = 0.02, significant)	-0.14 (P = 0.28)	-0.37 (P = 0.004, significant)	-0.22 (P = 0.08)	0.23 (P = 0.08)
Serum Potassium	-0.26 (P = 0.045, significant)	-0.10 (P = 0.45)	-0.19 (P = 0.14)	-0.02 (P > 0.8)	-0.21 (P = 0.10)	-0.14 (P = 0.28)	0.08 (P = 0.5)
Serum Albumin	-0.06 (P = 0.6)	0.04 (P > 0.7)	0.13 (P = 0.32)	-0.18 (P = 0.16)	0.14 (P = 0.28)	0.07 (P = 0.6)	0.16 (P = 0.22)
GFR	0.15 (P = 0.24)	-0.05 (P > 0.6)	0.33 (P = 0.01, significant)	0.04 (P > 0.7)	0.03 (P > 0.7)	0.04 (P > 0.7)	0.19 (P = 0.14)

## Discussion

In this study of 60 eyes, retinal nerve fibre layer (RNFL) thinning was associated with worsening chronic kidney disease (CKD). A significant reduction in inferior RNFL thickness was observed with advancing CKD stage, while temporal RNFL thickness showed a borderline significant difference. Mean retinal thickness and ganglion cell layer (GCL) thickness also progressively decreased from CKD stage 3 to stage 5.

Several studies have reported reductions in both retinal and RNFL thickness in patients with CKD. Paterson et al. demonstrated a significant reduction in total retinal thickness and inner retinal layer thickness with advancing CKD, particularly from stages 1-2 to stages 4-5 [11]. Majithia et al. reported significant RNFL and GC-IPL thinning in CKD patients with suboptimal kidney function and noted that reduced GFR was associated with thinner RNFL [15]. This association can be attributed to the fact that both the retina and the kidneys are highly vascular structures; therefore, systemic diseases such as CKD can result in microvascular changes affecting both organs.

In a prospective study, Yeung et al. evaluated longitudinal changes in peripapillary RNFL thickness in patients with CKD and demonstrated a significantly faster rate of RNFL thinning than in controls after adjusting for confounding factors. Furthermore, patients with advanced CKD (stages 4 and 5) exhibited more pronounced RNFL loss, predominantly in the superior and inferior quadrants, respectively [16].

Several mechanisms have been proposed to explain retinal and nerve fibre layer thinning in CKD, including retinal ischemia, chronic inflammation, oxidative stress, and involvement of the renin-angiotensin system (RAS) [17]. The RAS plays a key role in regulating blood pressure and fluid balance, and its dysregulation induces oxidative stress and inflammation, thereby affecting both renal and retinal tissues [18]. Consequently, renal dysfunction may lead to retinal microvascular changes that, in turn, affect the RNFL and GC-IPL layers [19].

Studies have also reported a greater impact of CKD on Indian eyes, with more pronounced thinning of the RNFL and GC-IPL. This is likely due to inherently thinner RNFL and GC-IPL thickness in Indian eyes, making them more susceptible to microvascular damage secondary to CKD [20]. Anchitha et al. demonstrated a negative correlation between retinal morphological parameters and CKD severity, with progressive disease associated with reduced macular thickness and volume [21].

Peng et al. evaluated the effect of blood pressure control on retinal microvasculature in patients with CKD. They found no significant correlation between retinal microvascular parameters and the duration of CKD or hypertension [22]. This finding is consistent with our study, in which no statistically significant changes in retinal parameters were observed when stratified by disease duration.

In our study, elevated HbA1c showed a positive correlation with increased retinal thickness and thicker inferior RNFL. Both hypernatremia and hyperkalemia were associated with reduced retinal thickness. With respect to renal function, higher GFR was positively correlated with lower RNFL thickness. Previous studies have also reported a positive correlation between HbA1c and central macular thickness [23]. However, in contrast to our findings, some studies have reported no association between HbA1c and RNFL thickness [24].

This study provides valuable insight into retinal structural changes and their association with biochemical alterations in patients with CKD. Correlating biochemical parameters with OCT findings provides a comprehensive overview of retinal involvement in CKD.

However, the relatively small sample size may limit the generalizability of the findings; therefore, larger studies are warranted. In addition, the cross-sectional design precludes establishing causal relationships; hence, longitudinal studies would be more appropriate.

Future research should include larger, multicenter cohorts and patients at earlier stages of CKD to determine whether retinal changes occur early in the disease course and whether early detection can help prevent ocular damage.

## Conclusion

This study demonstrated selective neuroretinal alterations in CKD patients, which become more pronounced with increasing disease severity. SD-OCT revealed significant thinning of the inferior RNFL and a reduction in retinal and ganglion cell thickness. Correlation between OCT parameters and biochemical markers such as HbA1c, electrolytes, and GFR highlights the influence of systemic factors on retinal structure. OCT, therefore, serves as a valuable tool for non-invasive screening, monitoring, and assessing disease progression in CKD.
